# 63 Effect of Gender on Risk of PTSD/Depression and Symptom Outcomes 30-Days Post-injury in Burn Patients

**DOI:** 10.1093/jbcr/irae036.055

**Published:** 2024-04-17

**Authors:** Yulia Gavrilova, Julia Donevant, Julia Ficalora, Steven A Kahn

**Affiliations:** South Carolina Burn Center / Medical University of South Carolina, Charleston, South Carolina; MUSC, Charleston, SC; South Carolina Burn Center / Medical University of South Carolina, Charleston, South Carolina; MUSC, Charleston, SC; South Carolina Burn Center / Medical University of South Carolina, Charleston, South Carolina; MUSC, Charleston, SC; South Carolina Burn Center / Medical University of South Carolina, Charleston, South Carolina; MUSC, Charleston, SC

## Abstract

**Introduction:**

Annually, nearly 45,000 people in the U.S. are hospitalized for burn injuries. Burn patients experience significant psychological burden during acute and post-acute burn treatment. Studies have examined the psychological sequelae of a traumatic burn injury (e.g., Acute Stress Disorder, Posttraumatic Stress Disorder [PTSD], Depression), however, many studies do not consider the role gender plays in burn recovery among burn survivors. This study aims to examine the differences between male and female burn survivors and their risk of developing PTSD and depression early on as well as their symptoms 30 days post-injury.

**Methods:**

Participants included 378 patients enrolled in the Burn Behavioral Health program at a regional Burn Center (Male: 66%; Female: 34%). The risk of PTSD and depression was assessed via the Injured Trauma Survivor Screen (ITSS) within the initial 30 days following a burn injury and the symptom outcome was assessed via the PTSD Checklist (PCL-5 Short Form) and the Patient Health Questionnaire (PHQ-8) 30 days post-injury.

**Results:**

Independent samples t-tests revealed that, compared to males, females reported statistically greater Total risk scores of developing PTSD/Depression (p=.011) early in the admission. A subscale analysis indicated that females reported greater risk scores on the Depression subscale (p=.007), but not on the PTSD subscale (p>.05). At 30-day follow-up, females reported greater scores on the PCL-5 (p=.023), but not on the PHQ-8 (p>.05). When TBSA was included as a covariate (ANCOVA test), gender remained a statistically significant predictor of the Total risk score (p=.002) and Depression subscale risk score (p < .001) during initial screening, and 30-day follow-up PTSD symptoms score (p=.011), while TBSA did not. TBSA (but not gender), on the other hand, had a statistically significant effect on the PTSD subscale score during initial screening (p=.007).

**Conclusions:**

Gender and TBSA play a role in an individual’s risk of developing PTSD and depression, with females being more at risk for depression early on and larger TBSA being a significant risk factor for PTSD early on regardless of gender. Contrary to risk results, females did not report higher depression scores 30 days post-injury, but they did report higher PTSD scores than males.

**Applicability of Research to Practice:**

This study expands our understanding of the effect gender has on the risk of developing PTSD/depression and symptom outcomes 30 days post-injury. Results suggest that gender is a significant predictor of these outcomes even when controlling for TBSA. TBSA plays a role in predicting the risk of developing PTSD, independent of gender. This highlights the significance of considering gender-specific differences in mental health risk among burn survivors, emphasizing the need for early screening and intervention, and recognizing the role of TBSA in predicting mental health outcomes, which can inform targeted support strategies for this patient population.

